# Ontogenic differences in sexual size dimorphism across four plover populations

**DOI:** 10.1111/ibi.12263

**Published:** 2015-04-23

**Authors:** Natalie Dos Remedios, Tamás Székely, Clemens Küpper, Patricia L. M. Lee, András Kosztolányi

**Affiliations:** ^1^ Department of Biology and BiochemistryUniversity of BathClaverton DownBathBA2 7AYUK; ^2^ NERC‐Biomolecular Analysis FacilityDepartment of Animal and Plant SciencesUniversity of SheffieldWestern BankSheffieldS10 2TNUK; ^3^ Centre for Integrative EcologySchool of Life and Environmental SciencesDeakin UniversityWarrnamboolVictoria3280Australia; ^4^ Department of BiosciencesCollege of ScienceSwansea UniversitySingleton ParkSwanseaSA2 8PPUK; ^5^ MTA‐DE ‘Lendület’ Behavioural Ecology Research GroupDepartment of Evolutionary ZoologyUniversity of DebrecenEgyetem tér 1.H‐4032DebrecenHungary

**Keywords:** Charadriiformes, development, growth, ontogeny, sexual size dimorphism, tarsus, waders

## Abstract

Sexual size dimorphism (SSD) among adults is commonly observed in animals and is considered to be adaptive. However, the ontogenic emergence of SSD, i.e. the timing of divergence in body size between males and females, has only recently received attention. It is widely acknowledged that the ontogeny of SSD may differ between species, but it remains unclear how variable the ontogeny of SSD is within species. Kentish Plovers *Charadrius alexandrinus* and Snowy Plovers *C. nivosus* are closely related wader species that exhibit similar, moderate (*c*. 4%), male‐biased adult SSD. To assess when SSD emerges we recorded tarsus length variation among 759 offspring in four populations of these species. Tarsus length of chicks was measured on the day of hatching and up to three times on recapture before fledging. In one population (Mexico, Snowy Plovers), males and females differed in size from the day of hatching, whereas growth rates differed between the sexes in two populations (Turkey and United Arab Emirates, both Kentish Plovers). In contrast, a fourth population (Cape Verde, Kentish Plovers) showed no significant SSD in juveniles. Our results suggest that adult SSD can emerge at different stages of development (prenatal, postnatal and post‐juvenile) in different populations of the same species. We discuss the proximate mechanisms that may underlie these developmental differences.

Sex differences in the body size of males and females are prevalent across the animal kingdom and their adaptive significance has been under investigation for over a century (Darwin 1871). Across taxa, the evolution of sexual size dimorphism (SSD) has been associated both with sexual selection for larger male body size (Székely *et al*. 2000, Bertin & Cézilly 2003, Lislevand *et al*. 2009) and with natural selection for larger female body size linked to increased fecundity (Blanckenhorn 2000, Blondel *et al*. 2002, Fairbairn 2007). However, among birds, body size is not always strongly related to fecundity (Serrano‐Meneses & Székely 2006, Lislevand *et al*. 2009) and the magnitude of SSD varies across bird species. To fully understand the evolution of SSD in birds, it is necessary to determine both how and when SSD develops among individuals at the proximate level (Price 1984).

Despite numerous studies focusing on patterns of adult SSD, research has only recently focused on the ontogenic development of SSD (Badyaev *et al*. 2001a, Blanckenhorn *et al*. 2007, Dietrich‐Bischoff *et al*. 2008, Hegyi *et al*. 2011, Klenovšek & Kryštufek 2013). Identifying exactly when morphological divergence between males and females starts to develop is of central importance in determining the underlying mechanisms (Cox & John‐Alder 2007, Stillwell *et al*. 2014). SSD may emerge during three main stages: (i) prenatally, due to sex differences in embryonic growth rates resulting either from intrinsic genetic differences (Godfrey & Farnsworth 1952, Sellier 2000) or from differences in maternal investment in male and female embryos (Müller *et al*. 2012, Helle *et al*. 2013); (ii) postnatally, due to sex differences in the rate or duration of growth of offspring (Leigh & Shea 1995, Blanckenhorn 2005, Hasumi 2010, Zhang & Liu 2013), which may be hormone‐mediated (e.g. linked to differential testosterone and activity levels; Klukowski *et al*. 2004, Cox *et al*. 2009); or (iii) post fledging, due either to sex differences in continued growth, or to mortality differences between juvenile or adult males and females in relation to body size (Badyaev *et al*. 2001a, Kersten & Brenninkmeijer 2008).

Comparative studies have revealed that variation in SSD often appears between closely related species. Even within a single species, variation in the extent of SSD can occur between different ecomorphs (Blanckenhorn *et al*. 2006, Dunham *et al*. 2013, Laiolo *et al*. 2013). For example, Blondel *et al*. (2002) found greater SSD in a mainland population of Blue Tits *Cyanistes caeruleus* than in an island population, where environmental stress was suggested to limit the size of the larger sex (males). Ontogenic factors are important in generating such population differences (Badyaev *et al*. 2001b). However, it remains unclear whether population differences in the ontogenic growth patterns of males and females might occur in species where adult SSD is consistent across populations.

Here, we investigate sex differences in body size during development in four plover populations: three populations of Kentish Plover *Charadrius alexandrinus* (Fig. 1) and one population of Snowy Plover *Charadrius nivosus*. The Snowy Plover and Kentish Plover are phenotypically similar and were long considered to be the same species (Hayman *et al*. 1988). Snowy Plovers are on average smaller than Kentish Plovers (tarsus length *c*. 25 and 29 mm, respectively; Küpper *et al*. 2009) but adults in these four populations exhibit very similar, moderate, male‐biased SSD (*c*. 1 mm, or 4%, difference in tarsus length, Küpper *et al*. 2009, T. Székely unpubl. data). Plover chicks are precocial, leaving the nest scrape a few hours after hatching, and feed for themselves throughout the postnatal growth period (Warriner *et al*. 1986, Székely & Williams 1994). In waders (notably plovers and sandpipers), sexual selection appears to play a major role in the evolution of sexual size dimorphism and is associated with male display behaviour during courtship (Székely *et al*. 2000, 2004). However, the proximate mechanisms responsible for the emergence of SSD in this group are still unclear.

**Figure 1:**
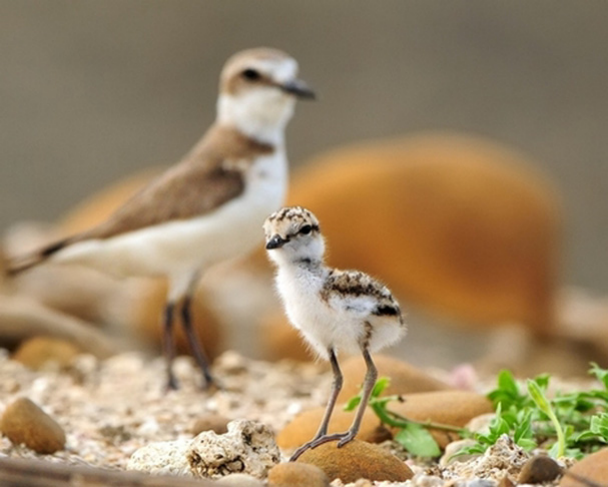
A Kentish Plover *Charadrius alexandrinus* chick attended by a parent. Photo: Su‐Shyue Liao.

We aim to assess when SSD develops ontogentically among plovers (prenatally, postnatally or post‐fledging), and whether SSD emerges during the same ontogenic period in different populations. We use tarsus length as a proxy for structural body size (Rising & Somers 1989, Senar & Pascual 1997), and compare hatchling size and growth up to fledging age (25 days) for males and females in each population.

## Methods

### Data collection

We collected hatchling size and growth data from four plover populations (Table 1). At Bahía de Ceuta, Mexico, we measured juvenile Snowy Plovers from May to July 2006–2009. Kentish Plover populations were investigated at Tuzla Lake, southern Turkey, from April to July 1996–1999 and in 2004; at Al Wathba Wetland Reserve, United Arab Emirates, between March and July 2005–2006; and on Maio Island, Cape Verde, from September to November 2008–2010.

**Table 1:** Summary data and growth parameters for chicks sampled across four plover populations. Parameters of the linear tarsus growth model (see text for details) are: (*a*) size at hatching (mm) and (*b*) growth rate (mm/day)

Species	Ceuta, Mexico	Tuzla, Turkey	Al Wathba, UAE	Maio, Cape Verde
	Snowy Plover	Kentish Plover	Kentish Plover	Kentish Plover
Location	23°54′N, 106°57′W	36°42′N, 35°03′E	24°15.5′N, 54°36.2′E	15°0.9′N, 23°12′W
No. of chicks measured on day of hatching, broods	262, 122	347, 144	70, 39	80, 51
Years of study	2006–2009	1996–1999, 2004	2005, 2006	2008–2010
No. of chicks measured between 1 and 25 days of age	126	115	33	41
Mean no. of captures per chick	2.2	2.3	2.0	1.6
Tarsus length (mm) at hatching*				
Males	17.60 ± 0.05	19.05 ± 0.06	18.52 ± 0.16	19.61 ± 0.18
Females	17.35 ± 0.05	18.92 ± 0.05	18.09 ± 0.17	19.47 ± 0.14
Tarsus growth parameters				
*a*	17.607	19.058	17.844	19.160
*b*	0.309	0.411	0.522	0.450
Fit of linear growth model: *r* ^2^	0.830	0.870	0.873	0.812

UAE, United Arab Emirates. *Mean ± se.

We followed standard protocols (Székely *et al*. 2008) to search for nests by observation from a mobile hide, from a car or on foot. We predicted hatch dates based on the floating stage of eggs in lukewarm water, and visited nests daily as the expected hatch dates approached. On the day of hatching, chicks were captured and marked with a single metal ring, and in most cases (70%) with an additional colour ring. The length of the right tarsus (tarsometatarsal bone) was measured to the nearest 0.1 mm. Structural measurements such as bill or wing length were not available for all populations as field workers aimed to minimize handling time for each chick and keep the stress for families at a minimum.

Measurement errors (% ME) were estimated for three of the four populations by comparison of variation within and between individuals. Within‐individual differences were based on comparison of right tarsus with left tarsus measurements using the formula (Bailey & Byrnes 1990):

$$\%\;\text{ME}=[s_{\mathrm{within}}^{2}/\left(s_{\mathrm{among}}^{2}+s_{\mathrm{within}}^{2}\right)]*100$$

where s^2^_within_ = MS_within_, and s^2^_among_ = (MS_among_ – MS_within_)/2. MS_within_ and MS_among_ are the within‐individual and between‐individual mean sum of squares of a Model II ANOVA. Measurement errors were estimated at 0.83% in Ceuta, 1.49% in Tuzla and 0.38% in Al Wathba, indicating that approximately 99% of the total variation was due to differences in tarsus length between birds rather than measurement error.

A droplet of blood (25 *μ*L) was taken for molecular sex‐typing, by puncturing the leg vein using a hypodermic needle (Oring *et al*. 1988). Blood was stored in Eppendorf tubes containing 1 mL of Queen's lysis buffer (Seutin *et al*. 1991). In total, 103 chicks (29 Ceuta, 33 Tuzla, 18 Al Wathba, 23 Maio) with known hatch date and age were not captured on the day of hatching, but were captured, measured and blood sampled subsequently. These chicks were not included in the hatchling size dataset but were included in growth calculations. We monitored broods up to the age of 25 days, as most chicks fledge at (or shortly after) this point (Székely & Cuthill 1999). We attempted to measure the tarsus length of chicks multiple times prior to fledging; chicks were recaptured during opportunistic encounters in the field and their tarsus length was recorded (Table 1). Where any pair of parents produced multiple broods within or between breeding seasons, we included chicks from only one brood to avoid pseudoreplication. The included brood was chosen to be the one with most chicks at hatching, or the first brood for which data were collected.

### Molecular sex‐typing

DNA was extracted from blood samples using an ammonium acetate extraction method (Nicholls *et al*. 2000, Richardson *et al*. 2001). For molecular sex‐typing, two markers were amplified via polymerase chain reaction (PCR): the first marker *Z‐002B* amplified homologous regions on the Z and W chromosome that differed in size (*Z‐002B*; Dawson 2007) and the second marker was W‐specific and hence amplified only in females (*Calex‐31*; Küpper *et al*. 2007). PCR‐amplification was conducted with a fluorescently labelled forward primer and unlabelled reverse primer on a DNA Engine Tetrad 2 Peltier Thermal Cycler under the following conditions: 95 °C for 15 min, followed by 35 cycles of 94 °C for 30 s, 56 °C for 90 s, 72 °C for 60 s, and a final extension of 60 °C for 30 min. PCR amplicons were visualized on an ABI 3730 automated DNA analyser. For the *Z‐002B* marker, the Z alleles did not differ in size (ZZ) so all males were homozygous, whereas in females (ZW) the Z and W alleles differed in size, appearing heterozygous (two differing hemizygous amplicons). PCR of *Calex‐31* amplified a W‐chromosome fragment in females, whereas in males no product was amplified. Using two different marker systems overcomes the problem of mis‐typing due to allelic dropout (Toouli *et al*. 2000) or Z polymorphism (Dawson *et al*. 2001, Dos Remedios *et al*. 2010). Alleles were scored using genemapper software version 4.1 (Applied Biosystems, MA, USA).

### Statistical analysis

#### Hatchling size

Because broods usually contained more than one chick (modal clutch size is three in Kentish and Snowy Plovers; Székely *et al*. 1994), we assessed variation in hatchling size using linear mixed models (LMMs) implemented in r version 3.0.2 (R Core Team 2013) using the package ‘lme4’ (version 1.0‐5; Bates *et al*. 2013). Brood identity was included as a random grouping structure to analyse sex differences in tarsus length of chicks on the day of hatching. We ran separate analyses for each population, rather than a single cross‐population model, as our aim was to monitor within‐population SSD development across two species that differed in body size (Küpper *et al*. 2009). We used standardized tarsus length (*z*‐values) as the response variable in LMMs for each population separately to provide a comparable measure of SSD across populations. Initial models included chick sex (two‐level factor), year (fixed factor) and hatch date (fixed covariate) together with their pairwise interactions. Because the timing and length of the breeding season differed between populations we also standardized hatch dates for each population using *z*‐values. Chick sex was retained in all models. For all other terms we applied stepwise model simplification, removing non‐significant terms one by one, until the ‘minimum model’ with lowest Akaike information criterion (AIC) value was reached for each population. Likelihood ratio tests were then carried out to estimate the significance of terms in the minimum models.

#### Chick growth

Early‐phase tarsus growth in plovers (aged 0–25 days) is well described by a linear equation (Székely & Cuthill 1999):

T=a+(b×D)

where *T* is tarsus length (in mm) at age *D* (in days), and *a* and *b* are estimated parameters (size at hatching and daily growth rate respectively; Table 1). Linear regression models were fitted for each population separately, including all tarsus length measurements of known‐age chicks captured at least once after the day of hatching (see Supporting Information Fig. S1 for plots of fitted lines).

To analyse the deviation of each data point (tarsus measurement) from the fitted linear regression models for chicks captured at any age (0–25 days old) in each population, standardized residuals were used as the response variable in LMMs, with chick identity nested within brood identity as a random grouping structure. Residuals were largely consistent for individuals measured multiple times independent of age (i.e. chicks remained larger or smaller than average with age, in 69–81% of cases in the four populations). In Ceuta, no chicks were recaptured in 2008, and therefore this year was excluded from the growth analyses for this population. Initial models included chick sex, year (fixed factors) and standardized hatch date (fixed covariate). We also included pairwise interactions between sex, year and hatch date and ran model simplifications as for hatchling size. Likelihood ratio tests were carried out to estimate the significance of terms in the minimum models. In two populations (Ceuta and Tuzla) several chicks took part in experiments in which eggs or chicks (shortly after hatching) were moved between nesting pairs. Therefore we ran separate model sets with either social or genetic brood ID as random factors. As the two model sets gave qualitatively the same results, we report only the results of models with social brood ID.

#### Cross‐population analyses

Sex differences in hatchling size and chick growth were tested independently for each study population (Methods above). However, the results of independent tests may not be directly comparable if datasets differ in sample size or if there is additional structure caused by other explanatory variables. Therefore, to statistically combine measures of effect for each independent test, to determine the presence and magnitude of an overall effect, and to measure the degree of heterogeneity in the data between locations, we implemented meta‐analytic methods in metawin version 2.0 (Rosenberg *et al*. 1999). The effect size estimates for sex differences in the final model in each population were calculated from likelihood ratio test Chi‐squared statistics using Fisher's *z*‐transformation. Negative values of Fisher's *z‐*transformation represent a negative effect, positive values represent a positive effect, and a zero value represents no effect. A cumulative effect size (grand mean) weighted by sample sizes was then calculated to represent the overall magnitude of the effect across populations. Heterogeneity across sample sets was estimated (*Q*_T_ values) to evaluate the likelihood that variance among effect sizes was greater than expected by sampling error. For both hatchling tarsus length and tarsus growth models, the ratio of the square root pooled variance to mean study variance (hatching ratio = 0.00; growth ratio = 0.81) indicated that cross‐population analyses were appropriate without the need for further grouping.

## Results

### Hatchling size

**Figure 2:**
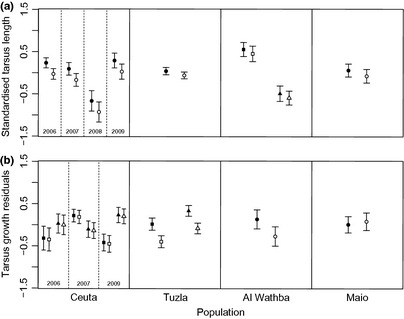
(a) Tarsus length on the day of hatching and (b) tarsus growth up to 25 days old for male and female chicks in four plover populations. Points represent predicted values (with standard error) from the best supported models (see Table 2). Separate plots are provided to visualize the effects of significant environmental variables: ‘year’, ‘hatch date’ and their interactions. Note that hatch date was a continuous variable in the analyses; however, it has been converted to a binary variable (early, late) to visualize interactions here. Males are represented by filled symbols, females by open symbols. Circles represent means for the entire season, squares for the early season and triangles for the late season.

In Ceuta, male chicks had significantly larger tarsi than females on the day of hatching (*β *= 0.263 ± 0.099 (se); Table 2). In the other three populations, no significant sex differences in hatchling size were identified, although in all cases a non‐significant trend emerged for larger male than female hatchlings (Fig. 2a). In Ceuta and Al Wathba, the tarsus length of hatchlings varied over time: in Ceuta, chicks hatching in 2008 had shorter tarsi than those hatching in other years of the study and in Al Wathba, tarsus length was shorter among those hatching later in the season (*β *= −0.631 ± 0.120; Fig. 2a).

**Table 2:** Tarsus length of plover chicks on the day of hatching and tarsus growth up to age 25 days. The significance of terms in ‘minimum’ linear mixed models was assessed by likelihood ratio tests

Response	Predictor	Ceuta, Mexico		Tuzla, Turkey		Al Wathba, UAE		Maio, Cape Verde	
		*χ* ^2^ (df)	*P*	*χ* ^2^ (df)	*P*	*χ* ^2^ (df)	*P*	*χ* ^2^ (df)	*P*
Hatchling tarsus length	Sex	6.94 (1)	0.008*	1.06 (1)	0.304	0.33 (1)	0.568	0.37 (1)	0.544
	Hatch date					21.07 (1)	<0.001*		
	Year	12.59 (3)	0.006*						
Tarsus growth residuals	Sex	0.66 (1)	0.418	6.67 (1)	0.010*	1.67 (1)	0.196	0.09 (1)	0.768
	Hatch date			4.26 (1)	0.039*				
	Year × hatch date	8.36 (2)	0.015*						

UAE, United Arab Emirates.

Values marked with * indicate significant effects (*P *≤* *0.05).

**Figure 3:**
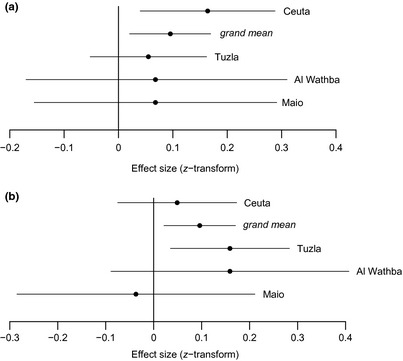
Mean effect sizes (Fisher's *z*‐transformation) with associated 95% confidence intervals for sex differences in (a) tarsus length at hatching and (b) tarsus growth across four plover populations. The *grand mean* represents the overall magnitude of effects across populations, weighted by sample sizes.

Meta‐analytic results supported the existence of population differences in SSD at hatching. For Ceuta, the effect size for tarsus length exceeded the grand mean (*Zr* = 0.164, var(*Zr*) = 0.004; grand mean 0.095; Fig. 3) in contrast to the relatively lower effect sizes of the other three populations. Furthermore, no significant heterogeneity among datasets emerged (*Q*_T_ = 1.874, 3 df, *P *=* *0.599).

### Chick growth

In Tuzla, male chicks grew faster than females (*β *= 0.386 ± 0.146; Table 2). In contrast, no significant sex differences were identified in Ceuta, Al Wathba or Maio, based on independent testing. In Tuzla, chicks that hatched later in the season grew faster than those hatching earlier on (*β *= 0.192 ± 0.089; Table 2) and there was also a trend for faster growth among later chicks in Ceuta in two of three study years (2006 and 2009; year × hatch date interaction; Table 2, Fig. 2b).

After correcting for sample size, meta‐analyses indicated that the magnitude of the effect size in Al Wathba (*Zr* = 0.159, var(*Zr*) = 0.016) was similar to that of Tuzla (*Zr* = 0.159, var(*Zr*) = 0.004; Fig. 3), suggesting a trend towards faster male than female growth in both populations, in contrast to Ceuta and Maio, where effect sizes were below the grand mean (0.096). No significant heterogeneity among datasets emerged (*Q*_T_ = 3.027, 3 df, *P *=* *0.388), suggesting that the variance among effect sizes was within the range expected due to sampling error alone.

## Discussion

Our results suggest that despite very similar SSD in adults (4%, Küpper *et al*. 2009, T. Székely unpubl. data), the ontogeny of SSD differs among populations in two closely related plover species (Fig. 2). In Ceuta, male Snowy Plover chicks were larger than females on the day of hatching, but subsequent growth rates until fledging (age 25 days) did not differ between the sexes. This suggests that SSD in the Ceuta population can largely be attributed to prenatal development. Conversely, in Tuzla, male and female Kentish Plover hatchlings did not differ significantly in size but male chicks grew faster than female chicks up to fledging, suggesting that SSD in this population developed largely postnatally. A similar effect was identified at Al Wathba (Kentish Plover) in cross‐population meta‐analyses (though non‐significant based on LMMs). Lastly, in the Maio Kentish Plover population, no significant sex differences in body size were identified among chicks at any stage, therefore the SSD previously observed among the adults of this population is more likely to have emerged after fledging due to either sex differences in growth rate, growth duration, or differential survival relative to size.

Although minor differences in sampling were present among populations, these sampling biases cannot explain the observed results. Despite sample sizes being lower for two of the four populations (Al Wathba, Maio; Table 1), meta‐analytic comparison of effect sizes enabled the identification of population differences in sex‐bias, and no significant heterogeneity emerged between datasets. Additionally, a larger proportion of recapture measurements was available for younger than older chicks (pre‐ rather than post‐12.5 days); however, the relative distribution of measurements by age was similar across populations (mean age at capture: Ceuta 5 days, Tuzla 7 days, Al Wathba 6 days, Maio 4 days).

In contrast to previous studies (Badyaev *et al*. 2001b, Blondel *et al*. 2002, Blanckenhorn *et al*. 2006, Laiolo *et al*. 2013), very similar levels of SSD were identified across populations of breeding adult plovers, but our results suggest this SSD emerges at different ontogenic stages in different populations. We suggest that multiple proximate mechanisms may therefore be involved, enabling divergence in body size at different stages even within a single species. Badyaev (2002) suggested that at any given age, the level of SSD would depend on the relative influence of age‐specific genetic effects, environmental effects, maternal effects and age‐specific epigenetic interactions. Whether SSD develops may depend on sex‐specific responses to the external environment as well as responses to internal selection pressures, for example differences in the functional coordination of developmental processes (Gebhardt‐Henrich & Richner 1998, Badyaev 2002, Klenovšek & Kryštufek 2013).

The three Kentish Plover populations studied are genetically similar and distinct from the Snowy Plovers (Küpper *et al*. 2009, 2012). Patterns of SSD ontogeny were consistent with genetic differences, as meta‐analyses suggested the two most closely related populations, Tuzla and Al Wathba, exhibited similar postnatal SSD development between 0 and 25 days of age. Environmental differences between populations may also affect sex‐specific development. Previous studies have reported context‐dependent SSD in relation to environmental variation. For example, variation in climate, food resources or parasite abundance can influence patterns of growth differently for males and females, leading to differences in the extent of adult SSD in closely related populations (Richner 1989, Cooch *et al*. 1996, Sheldon *et al*. 1998, Badyaev *et al*. 2001a, Blondel *et al*. 2002, Blanckenhorn *et al*. 2006, Hegyi *et al*. 2011, Stillwell *et al*. 2014). Whether sex differences emerge prenatally, postnatally or after fledging may depend on whether sex differences in growth are more pronounced under detrimental or favourable abiotic or biotic conditions.

Differences in ambient temperature have been reported among the four study populations (Ceuta 34.07 ± 8.46 °C; Tuzla 25.63 ± 2.88 °C; Al Wathba 35.22 ± 9.17 °C; Maio 29.31 ± 5.08 °C; Vincze *et al*. 2013). However, these differences are not consistent with the observed population differences in development of SSD among plovers, as Snowy Plovers from Ceuta and Kentish Plover chicks from Al Wathba hatched at very similar temperatures but showed different ontogenies for SSD. Furthermore, although overall tarsus length at hatching or tarsus growth varied significantly with hatch date and/or year in three of the four populations, no significant interactions emerged between seasonal variables and the sex of offspring, suggesting that within‐population environmental variation influenced both sexes equally.

One environmental factor that may be correlated with the observed population differences is locality: Maio (where no sex differences were identified among offspring) is an island location (Cape Verde, 625 km from mainland Africa), whereas the other three populations are in mainland regions. Offspring development in island populations is often slower than on the mainland (Andrews 1976, Higuchi & Momose 1981). One possibility is that males in Maio continue to grow beyond 25 days, causing SSD to develop later on. Our monitoring did not continue beyond 25 days due to the difficulties of recapturing fledged chicks. Although the tarsus length of fledglings was approaching that of adults across study populations (Küpper *et al*. 2009; T. Székely unpubl. data), to understand more fully how SSD develops it would be necessary to recapture and monitor growth of juvenile plovers beyond 25 days of age.

Alternatively, adult SSD may emerge through sex‐biased differences in migration patterns, dispersal or behavioural exclusion of larger or smaller individuals (Stamps 1993, Watkins 1996, Haenel & John‐Alder 2002, Cox & John‐Alder 2007). The reported levels of SSD were based upon breeding adults captured either on the nest or with chicks (Küpper *et al*. 2009, T. Székely unpubl. data). This may not provide a true representation of the body size distribution for the entire population. For example, it may be that smaller males do not breed in some populations such as Maio, and SSD may be reduced or absent in the population as a whole.

The patterns of sex‐biased offspring development identified within this study may have implications for population‐level evolutionary processes. Sex‐biases in body size can influence the relative mortality rates of males and females, and among bird species with only moderate SSD such as in plovers, the larger sex usually has the survival advantage (Bortolotti 1986, Oddie 2000, Hipkiss *et al*. 2002, Råberg *et al*. 2005, Rowland *et al*. 2007). Sex‐biased mortality can lead to biased adult sex ratios (ratio of males to females). There is increasing theoretical and empirical evidence for a role of adult sex ratio in the evolution of parental care and mating systems (Székely *et al*. 1999, Kokko & Jennions 2008, Jennions & Kokko 2010, Kosztolányi *et al*. 2011, Liker *et al*. 2013). This is of particular relevance among Kentish and Snowy Plovers, as extremely high variation has been reported in parental care strategies at the population level (Kosztolányi *et al*. 2009, Argüelles‐Tico 2011).

In summary, we present evidence that moderate male‐biased adult SSD may emerge at different stages of development (prenatal or postnatal) across closely related species and also across genetically similar populations of the same species. Further comparative cross‐population studies are needed to address how the ontogeny of SSD varies in relation to particular environmental conditions, and to determine the proximate mechanisms involved in driving variation in SSD ontogeny among populations.

We thank Istvan Szentirmai, Adam Lendvai, Thijs van Overveld, Medardo Cruz‐Lopez, Lydia Lozano‐Angulo, Iwan Fletcher and Araceli Argüelles‐Tico for helping with fieldwork. We also thank the staff at the wetland reserves at Al Wathba, Tuzla Lake, the Sea Turtle Program (PROTOMAR), Universidad Autónoma de Sinaloa and Salina Porto Ingles, Maio, for logistical support. We are grateful to Dan Ruthrauff, Jeroen Reneerkens and an anonymous reviewer for providing constructive comments that helped to improve the manuscript. This study was funded by an NERC studentship (No. NE/H525011/11) to T.S., and formed part of the PhD dissertation of N.d.R. Molecular sexing was conducted at NERC‐Biomolecular Analysis Facility at the University of Sheffield. We thank Deborah Dawson for supplying aliquots of the unpublished *Z‐002B* sex‐typing marker. C.K. was supported by a Marie Curie IEF postdoctoral fellowship. A.K. was supported by the Hungarian Scientific Research Fund (OTKA K81953) and the Hungarian Academy of Sciences (‘Lendület’ LP2012‐37/2012). Permits for fieldwork were provided by the national agencies in each country.

## Supporting information


**Figure S1.** Tarsus length of chicks monitored between hatching and fledging across four plover populations.
