# Supplementary material for: Ontogenic differences in sexual size dimorphism across four plover populations
Source: Ibis (Lond 1859). 2015 Apr 23;157(3):590–600. doi: 10.1111/ibi.12263 (PMC4957268; doi:10.1111/ibi.12263)
Supplement: Supplementary file 1 — Figure S1. Tarsus length of chicks monitored between hatching and fledging across four plover populations. [file IBI-157-590-s001.doc]

**Figure S1.** Tarsus length of chicks monitored between hatching and fledging across four plover populations: a) Ceuta, b) Tuzla, c) Al Wathba and d) Maio. For each population, the estimated linear growth line and 95% predicted intervals (± two standard deviations) are plotted based on ordinary least squares regression models (*r^2^* values are provided in Table 1).


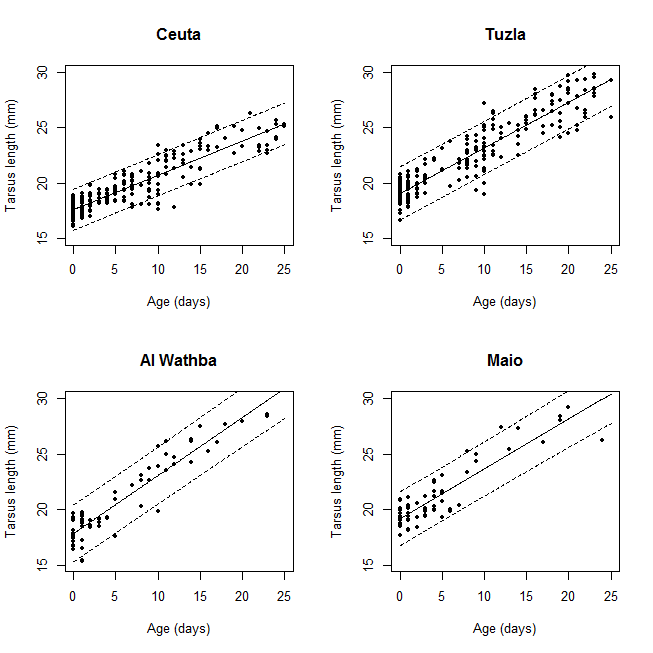

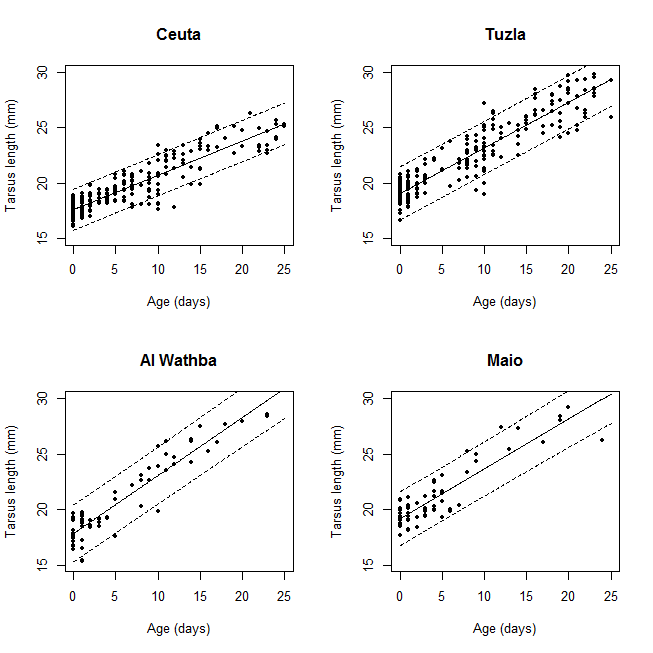


a)

b)

c)

d)
